# Genome Sequence of the Saprophytic Ascomycete *Epicoccum* *nigrum* Strain ICMP 19927, Isolated from New Zealand

**DOI:** 10.1128/genomeA.00557-17

**Published:** 2017-06-15

**Authors:** Mikhail Fokin, Damien Fleetwood, Bevan S. Weir, Silas Villas-Boas

**Affiliations:** a School of Biological Sciences, University of Auckland, Auckland, New Zealand; b Biotelliga Limited, Auckland, New Zealand; c Landcare Research, Auckland, New Zealand

## Abstract

*Epicoccum nigrum* is a common mitosporic fungus of the Didymellaceae (Ascomycota) family known for the production of numerous secondary metabolites. Here, we present the 34.7-Mbp draft genome sequence of strain ICMP 19927 assembled from a range of short-insert and long-insert Illumina libraries.

## GENOME ANNOUNCEMENT

*Epicoccum* *nigrum* Link 1816 (synonym *E. purpurascens* Ehrenb. 1818) is primarily a saprophytic species which has been isolated from virtually all possible terrestrial and marine substrates worldwide (1–3). Substantial intraspecific morphological and genetic diversity was reported for this species, suggesting the presence of at least two divergent clades (4–6). It is also well known for numerous secondary metabolites, with pigments isolated more than a century ago (7–10). The antifungal activities of some of these compounds led to attempts to develop biological control products based on *E. nigrum* mycelia, spores, and metabolites (11–14). Full-genome data will facilitate the characterization of secondary metabolite pathways and contribute to the clarification of taxonomic issues.

The *E. nigrum* strain ICMP 19927 was isolated in 2007 as likely airborne contamination of agar plates at Auckland University city campus (New Zealand) and deposited in the International Collection of Microorganisms from Plants (ICMP). A single spore culture was grown on Czapek yeast extract agar plates at 25°C for 12 days. DNA was extracted and purified by G-100 Qiagen genomic columns, according to the manufacturer’s instructions for tissues, and submitted to New Zealand Genomics Limited (NZGL) for library preparation and sequencing. Five different libraries were constructed: two TruSeq Nano libraries with insert sizes of 350 and 550 bp and three Nextera mate-pair libraries with insert sizes of 3, 9, and 12 kbp. All libraries except the TruSeq Nano 350 bp were sequenced on a single MiSeq lane, while the TruSeq Nano 350-bp library was sequenced separately on the HiSeq 2500. In total, 20.5 M paired 300-bp reads and 11.3 M paired 125-bp reads were received.

NextClip version 1.3 (15) and Trimmomatic 0.35 (16) packages were used to remove external and internal adapters and for quality trimming. The genome sequence was assembled by SPAdes 3.10.0 (17) with a maximal k-mer size of 99 bp and analyzed by QUAST 4.5 (18). The assembly consists of 1,645 scaffolds, of which 227 scaffolds are larger than 1 kb, with a total length of 34.7 Mbp, including the mitochondrial sequence assembled as a single contig. The longest scaffold reached 4.96 Mbp, the *N*_50_ length was 1.37 Mbp, and the *L*_50_ was 7. The completeness of the assembly was assessed by BUSCO 2.0 (19) and showed the presence of 99.1% of Ascomycota single-orthologous genes and 99.6% of the Pezizomycotina set.

The genome was annotated using the funannotate pipeline (version 0.6.0), which includes repeat masking, training, gene prediction, and annotation steps. In total, 12,049 protein-coding gene models were annotated, which is consistent with JGI-derived predictions for other Didymellaceae species but significantly higher than the 9,495 genes predicted for pathogenic *Epicoccum sorghinum* (20, 21). This annotated genome sequence is only the fourth from the Didymellaceae family and will contribute to a better understanding of the phylogenetic diversity and metabolic potential of *E. nigrum*.

### Accession number(s).

This whole-genome shotgun project has been deposited in DDBJ/ENA/GenBank under the accession no. NCTX00000000. The version described in this paper is the first version, NCTX01000000.
